# Discolored Ureteral Stents: Findings in Urinalysis and Urine Culture

**DOI:** 10.1371/journal.pone.0122984

**Published:** 2015-04-07

**Authors:** Takashi Kawahara, Hiroshi Miyamoto, Hiroki Ito, Hideyuki Terao, Hiroji Uemura, Yoshinobu Kubota, Junichi Matsuzaki

**Affiliations:** 1 Department of Urology, Yokohama City University Graduate School of Medicine, Yokohama, Kanagawa, Japan; 2 Department of Urology, Ohguchi Higashi General Hospital, Yokohama, Kanagawa, Japan; 3 Departments of Pathology and Urology, Johns Hopkins University School of Medicine, Baltimore, MD, United States of America; Cedars-Sinai Medical Center, UNITED STATES

## Abstract

**Objective:**

Discolored ureteral stents are sometimes encountered in daily clinical practice; however, the mechanism(s) underlying the development of discolored ureteral stents remain unknown. In this study, we retrospectively analyzed the characteristics of discolored ureteral stents based on the results of a urinalysis and urine culture.

**Materials & Methods:**

We identified a total of 26 patients with discolored ureteral stents and compared the findings in the urinalyses and urine culture in 21 discolored versus 45 non-colored ureteral stents.

**Results:**

The median and mean (±SD) duration of stenting time was 78.0 and 81.3 (± 21.3) days for the discolored ureteral stents and 69.0 and 74.9 (± 19.8) days for the non-colored ureteral stents, respectively (P = 0.25). The discolored ureteral stents were associated with a higher mean urine pH than the non-colored ureteral stents (mean: 6.4 vs 6.0, P< 0.05). There were no significant differences between the two groups in the RBC (P = 0.51) and WBC (P = 0.35) counts in the urinalyses. In addition, the rate of a positive culture in the patients with discolored stents [20 of 21 (95.2%)] was significantly (P <0.01) higher than that observed in the patients with non-colored ureteral stents [33 of 45 (73.3%)].

**Conclusions:**

In this study, the subjects with discolored ureteral stents showed a significantly higher likelihood of having a positive urine culture and also demonstrated higher pH values in the urinalyses. However, no clear cut-off point to predict discoloration was indicated.

## Introduction

In 1967, Zimskind *et al*. first reported the use of silicon ureteral splints to relieve ureteral obstruction cystoscopically [1]. Since then, ureteral stenting has become a fundamental part of various urological procedures, including both open and endoscopic ureteral surgery, as well as the management of obstructing ureteral calculi, ureteral stricture, ureteropelvic obstruction and retroperitoneal tumors or fibrosis or after open or endoscopic ureteral surgery [2,3]. On the other hand, serious complications of ureteral stenting continue to occur, including encrustation, incrustation, migration and fragmentation [2–9].

We previously analyzed 330 retrieved ureteral stents and reported that ureteral stent encrustation correlates with the indwelling time.[2] This result also supports the findings of a previous report published two decades ago by el-Faqih *et al*.[10] Moreover, there was a tendency for more discolored ureteral stents (black coloring) to be associated with encrustation than non-colored ureteral stents.

Discolored ureteral stents are sometimes encountered in daily clinical practice; however, the mechanism(s) underlying the development of such discoloration remain unknown[11,12]. In this study, we retrospectively analyzed data for the results of urinalyses and urine culture in patients with discolored ureteral stents.

## Patients and Methods

### Patient characteristics

This study was approved by the review board of Kanagawa Prefecture Medical Association (H25-3169). All ureteral stents were retrieved at Ohguchi Higashi General Hospital (Yokohama, Kanagawa, Japan), and written informed consent was obtained from all patients. From June 2010 to February 2011, a total of 330 ureteral stents were retrieved, as reported in our previous study[2]. Among these patients, 13 ureteral stents were heavily discolored and black in color. An additional 13 patients whose ureteral stents were heavily discolored were identified between February 2011 and September 2012, and non-colored ureteral stents recovered during the same period were used for comparison. All patients had stone disease; the background factors and characteristics of the patients are shown in Table 1. In agreement with our previous results, we found that the indwelling time was significantly longer in the patients with discolored ureteral stents than in those with non-colored ureteral stents (mean: 84.6 vs 61.9 days, p<0.05)[2]. Therefore, we compared the two groups (normal/discolored) with similar indwelling times, as shown in Table 2 (mean: 81.3 vs 74.9 days, p = 0.25). In order to ensure more homogenous patient background factors, subjects in whom ureteral stents were inserted as a consequence of ureteroscopic procedures were excluded from the current study due to the short indwelling time. The patient background data included age, sex, side of insertion and indwelling time (Tables 1 and 2).

**Table 1:** Patient characteristics and results of the urinalyses and urine cultures in all patients.

	**Discolored (Coloring to black) (n = 26)**			**Non-color (n = 69)**			**total**	
	**during indwelling stent**	**obtained at time separate from indwelling stent**	**all**	**during indwelling stent**	**obtained at time separate from indwelling stent**	**all**		***p***
**No of Pts.**	14	12	26	22	47	69	95	0.651
**Age**	56 (53.9±19.4)	60.5 (63.3±13.9)	59 (58.2±17.4)	54 (56.4±16.1)	58 (57.9±11.8)	58 (57.6±13.2)	59 (59.4±14.6)	<0.01
**Sex**								
Male	2 (14.3%)	5 (41.7%)	7 (26.9%)	11 (50.0%)	29 (61.7%)	40 (58.0%)	47 (49.5%)	<0.01
Female	12 (85.7%)	7 (58.3%)	19 (73.1%)	11 (50.0%)	18 (38.3%)	29 (42.0%)	48 (50.5%)	
**Side**								
Rt	4 (28.6%)	5 (41.7%)	9 (34.6%)	8 (36.4%)	26 (55.3%)	34 (49.3%)	43 (45.3%)	0.20
Lt	10 (71.4%)	7 (58.3%)	17(65.4%)	14 (63.6%)	21 (44.7%)	35 (50.7%)	52 (54.7%)	
**Indwelling time (median, mean±SD)**	69 (67.3±23.6)	81 (104.8±86.8)	75.5 (84.6±63.0)	65.5 (71.3±30.4)	56 (57.4±19.6)	61 (61.9±24.2)	64 (68.1±39.8)	<0.05
**(range; days)**	(28–103)	(30–365)	(28–365)	(29–143)	(29–101)	(29–143)	(28–365)	
**RBC**								
0+	3 (21.4%)	2 (16.7%)	5 (19.2%)	3 (14.3%)	21 (44.7%)	24 (34.8%)	29 (30.5%)	0.742
1+	1 (7.1%)	3 (25.0%)	4 (15.4%)	4 (19.0%)	10 (21.3%)	14 (20.3%)	18 (18.9%)	
2+	3 (21.4%)	5 (41.7%)	8 (30.8%)	2 (9.1%)	5 (10.6%)	7 (10.1%)	15 (15.8%)	
3+	7 (50.0%)	2 (16.7%)	9 (34.6%)	13 (61.9%)	11 (23.4%)	24 (34.8%)	33 (34.7%)	
**WBC**								
0+	4 (28.6%)	8 (66.7%)	12 (46.2%)	5 (23.8%)	29 (61.7%)	34 (49.3%)	46 (48.4%)	0.282
1+	1 (7.1%)	0 (0.0%)	1 (3.8%)	8 (39.15)	7 (14.9%)	15 (21.7%)	16 (16.8%)	
2+	0 (0.0%)	1 (8.3%)	1 (3.8%)	0 (0.0%)	3 (6.4%)	3 (4.3%)	4 (4.2%)	
3+	9 (64.3%)	3 (25.0%)	12 (46.2%)	9 (42.9%)	8 (17.0%)	17 (24.6%)	29 (30.5%)	
**pH**								
<7.0	9 (64.3%)	9 (75.0%)	18 (69.2%)	15 (71.4%)	44 (93.6%)	59 (85.5%)	77 (81.1%)	0.203
7.0–7.9	5 (35.7%)	1 (8.3%)	6 (23.1%)	5 (23.8%)	3 (6.4%)	8 (11.6%)	14 (14.7%)	
>8.0	0 (0.0%)	2 (16.7%)	2 (7.7%)	1 (4.5%)	0 (0.0%)	1 (1.4%)	3 (3.2%)	
Unknown	0 (0.0%)	0 (0.0%)	0 (0.0%)	1 (4.5%)	0 (0.0%)	1 (1.4%)	1 (1.1%)	
**Culture**								
E.coli	3 (21.4%)	5 (41.7%)	8 (30.8%)	7 (33.3%)	11 (23.4%)	18 (26.1%)	26 (27.4%)	
Klebsiella	4 (28.6%)	3 (25.0%)	7 (26.9%)	0 (0.0%)	13 (27.7%)	13 (18.8%)	20 (21.1%)	
Pseudomonas	2 (14.3%)	0 (0.0%)	2 (7.7%)	1 (4.5%)	8 (17.0%)	9 (13.0%)	11 (11.6%)	
Candida	1 (7.1%)	1 (8.3%)	2 (7.7%)	3 (14.3%)	0 (0.0%)	3 (4.3%)	5 (5.3%)	
Enterobacter	1 (7.1%)	1 (8.3%)	2 (7.7%)	1 (4.5%)	2 (4.3%)	3 (4.3%)	5 (5.3%)	
Staphylococcus	0 (0.0%)	0 (0.0%)	0 (0.0%)	2 (9.1%)	2 (4.3%)	4 (5.8%)	4 (4.2%)	
E faecalis	1 (7.1%)	0 (0.0%)	1 (3.8%)	0 (0.0%)	2 (4.3%)	2 (29.0%)	3 (3.2%)	
Acinetobacter	0 (0.0%)	2 (16.7%)	2 (7.7%)	0 (0.0%)	1 (2.1%)	1 (1.4%)	3 (3.2%)	
Citrobacter	2 (14.3%)	0 (0.0%)	2 (7.7%)	0 (0.0%)	1 (2.1%)	1 (1.4%)	3 (3.2%)	
Serratia	0 (0.0%)	0 (0.0%)	0 (0.0%)	2 (9.1%)	1 (2.1%)	3 (4.3%)	3 (3.2%)	
Corynebacterium	2 (14.3%)	0 (0.0%)	2 (7.7%)	0 (0.0%)	0 (0.0%)	0 (0.0%)	2 (2.1%)	
Enterococcus	0 (0.0%)	0 (0.0%)	0 (0.0%)	2 (9.1%)	0 (0.0%)	2 (29.0%)	2 (2.1%)	
Morganella morganii	1 (7.1%)	0 (0.0%)	1 (3.8%)	0 (0.0%)	0 (0.0%)	0 (0.0%)	1(1.1%)	
E cloacae	0 (0.0%)	0 (0.0%)	0 (0.0%)	0 (0.0%)	1 (2.1%)	1 (1.4%)	1(1.1%)	
St agaracture	0 (0.0%)	0 (0.0%)	0 (0.0%)	0 (0.0%)	1 (2.1%)	1 (1.4%)	1(1.1%)	
Negative	0 (0.0%)	1 (8.3%)	1 (3.8%)	4 (19.0%)	15 (31.95)	19 (27.5%)	20 (21.1%)	
**Positive Culture**	100.0%	91.6%	96.1%	81.8%	68.1%	72.5%	78.9%	0.101

**Table 2:** Patient characteristics and results of the urinalyses and urine cultures in the subjects with similar indwelling times for the ureteral stents.

	**Discolored (coloring to black)**	**Non-color**	**Total**	***p***
**No of Pts.**	21	45	66	
**Age**	59.9 (60.2+-3.6)	60.5 (59.2+-2.3)	60.2 (59.5+-1.9)	0.83
**Sex**				
Male	6 (28.6%)	27 (60.0%)	33 (50.0%)	0.038
Female	15 (71.4%)	18 (40.0%)	33 (50.0%)	0.038
**Side**				
Rt	5 (23.8%)	23 (51.1%)	28 (42.4%)	0.068
Lt	16 (76.2%)	22 (48.9%)	38 (57.6%)	
**Indwelling time: median, (mean±SD)**	78.0 (81.3+-4.6)	69 (74.9+-2.9)	71.5 (76.9+-2.5)	0.25
**(range:days)**	(51–124)	(50–143)	(50–143)	
**RBC**				
0+	4 (19.0%)	14 (31.1%)	18 (27.3%)	0.509
1+	4 (19.0%)	9 (20.0%)	13 (19.7%)	
2+	5 (23.8%)	5 (11.1%)	10 (15.2%)	
3+	8 (38.1%)	15 (33.3%)	23 (34.8%)	
**WBC**				
0+	9 (42.9%)	20 (44.4%)	29 (43.9%)	0.351
1+	1 (4.8%)	9 (20.0%)	10 (15.1%)	
2+	1 (4.8%)	2 (4.4%)	3 (4.5%)	
3+	10 (47.6%)	14 (31.1%)	24 (36.4%)	
**pH**	6.5 (6.4+-0.2)	6 (6.0+-0.1)	6 (6.1+-0.1)	<0.05
<7.0	13 (61.9%)	38 (84.4%)	51 (77.3%)	0.108
7.0–7.9	6 (28.6%)	6 (13.3%)	12 (18.2%)	
>8.0	2 (9.5%)	1 (2.2%)	3 (4.5%)	
**Culture**				
E.coli	7 (33.3%)	13 (28.9%)	20 (30.3%)	
Klebsiella	5 (23.8%)	8 (17.8%)	13 (19.7%)	
Pseudomonas	2 (9.5%)	5 (11.1%)	7 (10.6%)	
Staphylococcus	1 (4.8%)	4 (8.9%)	5 (7.6%)	
Candida	1 (4.8%)	2 (4.4%)	3 (4.5%)	
Enterobacter	2 (9.5%)	1 (2.2%)	3 (4.5%)	
Serratia	0 (0.0%)	3 (6.7%)	3 (4.5%)	
Acinetobacter	1 (4.8%)	1 (2.2%)	2 (3.0%)	
Corynebacterium	2 (9.5%)	0 (0.0%)	2 (3.0%)	
Enterococcus	0 (0.0%)	2 (4.4%)	2 (3.0%)	
E faecalis	0 (0.0%)	1 (2.2%)	1 (1.5%)	
Citrobacter	1 (4.8%)	0 (0.0%)	1 (1.5%)	
Morganella morganii	1 (4.8%)	0 (0.0%)	1 (1.5%)	
E cloacae	0 (0.0%)	1 (2.2%)	1 (1.5%)	
Negative	1 (4.8%)	12 (26.7%)	13 (19.7%)	
**Positive Culture**	95.20%	73.3%	80.30%	<0.01

### Urinalysis and urine culture

All urinalyses and urine cultures were performed before inserting/exchanging the ureteral stent and without the use of antibiotics. Because roughly half of the patients had previously been stented, we compared the results of the urinalyses and urine cultures separately for data obtained under conditions with and without indwelling stent placement.

### Coloring

**Fig 1:**
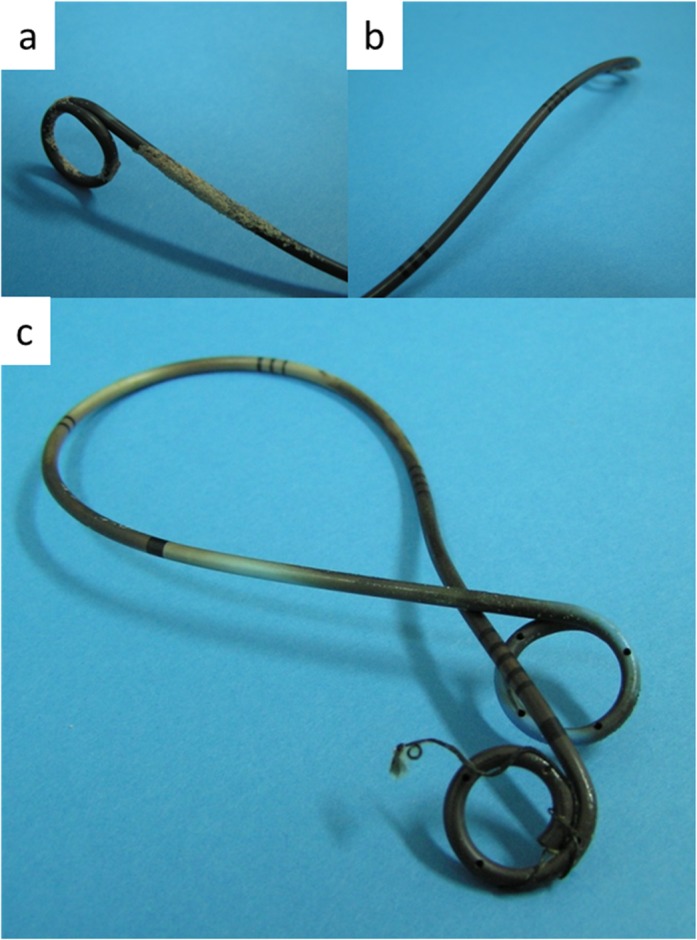
Discolored (black) ureteral stents. Discoloration in the (a) upper, (b) distal and (c) entire area of the stent.

The color of the ureteral stents was evaluated in the same manner as that described in our previous reports[2]. Briefly, we classified the stents as being either non-colored or discolored (black or brown) (Fig 1). If at least a quarter of the stent was brown, we classified the stent as “brown,” and if at least a quarter of the stent was black, we considered the stent to be “black.” Because our determinations had a tendency to be subjective, we compared only “black” and “non-colored” stents in this study [2,11,12]. All coloring grades were confirmed individually by at least two urologists.

### Statistical analysis

The numerical data were compared using Student’s *t*-test. The differences between two groups were compared according to a one-factor ANOVA. A *P* value of ≤ 0.05 was considered to be significant.

## Results

During the same period, 26 discolored (black) and 69 non-colored ureteral stents were identified. The median (mean ± SD) indwelling time was 75.5 (84.6 ± 63.0) days for the discolored stents and 61.0 (61.9 ± 24.2) days for the non-colored stents (p = 0.01) (Table 1). In order to match the patients’ background factors, we analyzed cases with an indwelling time of 50 to 143 days in each group. Ultimately, 21 discolored ureteral stents and 45 non-colored ureteral stents with similar indwelling times were compared. The median (mean ± SD) indwelling ureteral stenting time was 78.0 (81.3 ± 21.3) days and 69.0 (74.0 ± 19.8) days in the discolored (coloring black) and non-colored stent groups, respectively (p = 0.25). In the cohort with discolored ureteral stents, 11 patients (52.4%) with stents had data for both a urinalysis and urine culture, while the remaining 10 patients (47.6%) without stents had such data. Similarly, among the 45 control cases, 17 (37.8%) and 28 (62.2%) subjects had data for a urinalysis and urine culture, with or without stents, respectively. The patient age and treated side were not significantly different between the groups (Table 2), whereas discolored ureteral stents were seen significantly more often in females.

**Fig 2:**
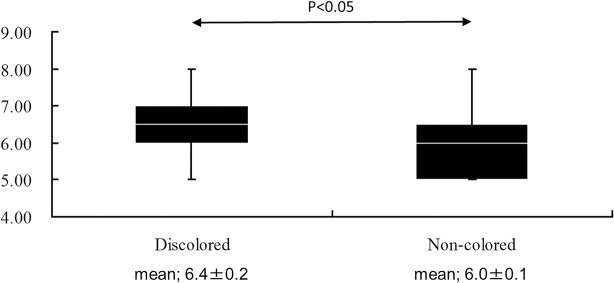
Urine pH in the patients with discolored vs non-colored ureteral stents.

The results of the urinalyses and urine cultures are also shown in Table 2. The patients with discolored ureteral stents showed a higher mean urine pH than those with non-colored ureteral stents (mean; 6.4 vs 6.o, p<0.05) (Fig 2). In contrast, there were no significant differences in the RBC (p = 0.509) and WBC (p = 0.351) counts on the urinalyses between the two groups. Fifty-three (80.3%) of 66 patients had positive urine cultures. The urine cultures in the patients with discolored ureteral stents were significantly more likely to be positive for bacteria (all: 95.2%; with stents: 100.0%; without stents: 90.0%) than those in the patients with non-colored ureteral stents (all: 73.3%; with stents: 82.4%; without stents: 67.9%), (p <0.01, 0.11 and 0.08, respectively). However, no differences were observed in the distribution of specimens positive for bacteria.

## Discussion

We previously demonstrated that ureteral stent encrustation correlates with the indwelling time and that discolored stents more frequently tend to be encrusted [2]. In the present study, the patients with discolored ureteral stents were also shown to have a longer indwelling time, supporting previous findings[10]. More importantly, our current analysis showed that the patients with discolored ureteral stents had significantly higher rates of positive urine cultures and higher pH values on the urinalyses compared to that observed in the patients with non-colored ureteral stents. Discolored ureteral stents are frequently detected, whereas few urethral balloon catheters show heavily black discoloration. We speculate that this difference is due to the opacity of ureteral stents, which makes it easy to visualize the stents on X-ray films [2].

Discolored ureteral stents are generally found in patients exhibiting positive urine cultures or those with a history of urinary tract infection. Our data support this observation, although most of the patients with non-colored ureteral stents in this study also showed a high frequency of infectious conditions, and all of our patients had urinary stones, which may have induced infection. Supporting this possibility, the types of bacteria distributed in the urine were similar between the two groups. It is therefore difficult to speculate as to the cause of the discolored ureteral stents in our patient population.

Discolored ureteral stents have been shown to more likely be encrusted [2]. However, neither encrustation nor incrustation are necessarily related to stent discoloration, but rather may reflect the condition of the urine. In general, there are several reasons for encrustation, including stone disease, urinary sepsis, chemotherapy, pregnancy, chronic renal failure and metabolic or congenital abnormalities, in addition to the indwelling time of the stent, each of which may have an effect on discoloration [2,13].

With regard to encrustation, the incidence of this finding may be affected by surface properties, such as roughness and irregularity. While the actual mechanism of encrustation is multi-faceted, the absorption of proteins on the stent is required to start the process.[2,14–17] The development of encrustation in patients with infected urine is therefore the result of the deposition of organic components in the urine onto the surface of the biomaterial that subsequently crystalize and become incorporated into the bacterial biofilm layer. The bacteria continue to grow and form a community, thus allowing for the production of bacterial urease, which in turn degrades urea and raises the urinary pH. The increased pH level then attracts calcium and magnesium ions to the biofilm matrix, leading to crystal formation.[16,17] In the current study, the patients with discolored ureteral stents showed significantly higher pH values on the urinalyses than did those with non-colored ureteral stents. Therefore, discoloration may precede encrustation.

It is important to emphasize that discoloration itself does not have any apparent adverse effects, and the most serious complication associated with ureteral stents is the inability to remove the ureteral stent due to heavy encrustation. Therefore, discoloration has the potential to be used as a new biomarker of encrustation. In addition, the presence of discoloration may indicate the appropriate indwelling time to change or remove the ureteral stent.

The current study has limitations that should be kept in mind when interpreting the results. First, this was a retrospective observational study. Second, discoloration, urinary tract infection and encrustation all arise as a result of various factors, including water intake, underlying illnesses, the presence of ureteral stones, etc. [2] Accordingly, our results may not reflect the cause of discoloration in all cases. Furthermore, in this study, none of the patients had metabolic stone disease, including that associated with uric acid, struvite or cysteine. Because the incidence of these stone was low, we did not find any correlations between discoloration and the chemical composition of the stones. At a minimum, we believe our findings to be meaningful when limited to patients with stone disease. Third, we assessed only patients with stone disease. During the course of treatment for stones, the duration of indwelling ureteral stenting is not long. Hence, further research is needed to assess the characteristics of ureteral stricture in these cases.

In conclusion, we herein provided the first findings of urinalyses and urine cultures in patients with discolored ureteral stents compared with that observed in a matched group of subjects with non-colored ureteral stents. Consequently, the patients with discolored ureteral stents showed significantly higher positive urine culture results and higher pH values on the urinalyses; however, no clear cut-off point for suspecting discoloration was indicated. Further research, including basic and clinical studies, is therefore needed to further elucidate the cause(s) of discoloration of ureteral stents and determine the potential use of discoloration as a biomarker for the risk of encrustation.
